# A surgical method for continuous intraportal infusion of gut microbial metabolites in mice

**DOI:** 10.1172/jci.insight.145607

**Published:** 2021-05-10

**Authors:** Danny Orabi, Lucas J. Osborn, Kevin Fung, William Massey, Anthony J. Horak, Federico Aucejo, Ibrahim Choucair, Beckey DeLucia, Zeneng Wang, Jan Claesen, J. Mark Brown

**Affiliations:** 1 Department of Cardiovascular and Metabolic Sciences and; 2 Center for Microbiome and Human Health, Lerner Research Institute of the Cleveland Clinic, Cleveland, Ohio, USA.; 3 Department of Molecular Medicine, Cleveland Clinic Lerner College of Medicine of Case Western Reserve University, Cleveland, Ohio, USA.; 4 Department of General Surgery, Cleveland Clinic, Cleveland, Ohio, USA.

**Keywords:** Metabolism, Microbiology, Molecular biology, Surgery

## Abstract

Gut microbe–derived metabolites influence human physiology and disease. However, establishing mechanistic links between gut microbial metabolites and disease pathogenesis in animal models remains challenging. The major route of absorption for microbe-derived small molecules is venous drainage via the portal vein to the liver. In the event of presystemic hepatic metabolism, the route of metabolite administration becomes critical. To our knowledge, we describe here a novel portal vein cannulation technique using a s.c. implanted osmotic pump to achieve continuous portal vein infusion in mice. We first administered the microbial metabolite trimethylamine (TMA) over 4 weeks, during which increased peripheral plasma levels of TMA and its host liver-derived cometabolite, trimethylamine-*N*-oxide, were observed when compared with a vehicle control. Next, 4-hydroxyphenylacetic acid (4-HPAA), a microbial metabolite that undergoes extensive presystemic hepatic metabolism, was administered intraportally to examine effects on hepatic gene expression. As expected, hepatic levels of 4-HPAA were elevated when compared with the control group while peripheral plasma 4-HPAA levels remained the same. Moreover, significant changes in the hepatic transcriptome were revealed by an unbiased RNA-Seq approach. Collectively, to our knowledge this work describes a novel method for administering gut microbe–derived metabolites via the portal vein, mimicking their physiologic delivery in vivo.

## Graphical abstract

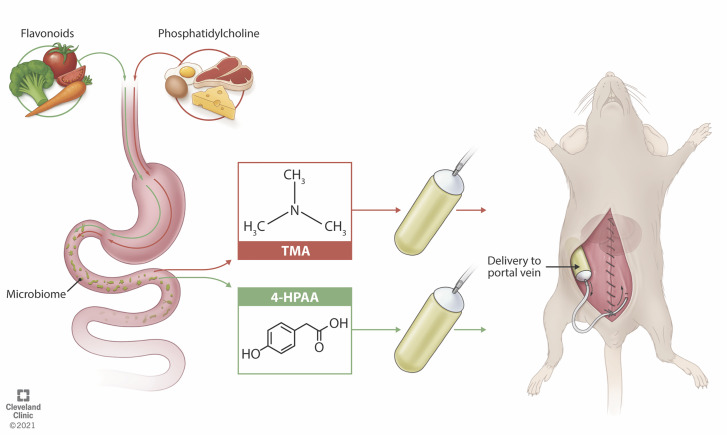

## Introduction

Recent efforts have underscored the importance of the gut microbial community as a meta-endocrine organ, impacting host physiology through systemic delivery of gut-microbial metabolites (1). Microbial metabolites are first delivered to the liver via the portal vein, following venous drainage of the gastrointestinal tract. This route of absorption is often crucial, allowing the liver to biotransform these molecules prior to entering the peripheral circulation. Microbial metabolites are frequently studied in animal models by incorporation into diet or drinking water. This method falls short as inconsistent oral intake, inconsistent gastrointestinal absorption, and further modification of the metabolite by gut microbes yield imprecise levels of drug delivery. In efforts to overcome this, the physiological impact of microbial metabolites is often studied by intermittent exogenous administration of a metabolite in a nonphysiologically relevant manner, such as i.v. injection, i.p. injection, or s.c. administration, all placing a relatively large proportion of the metabolite directly into the peripheral circulation. Although these approaches can effectively raise circulating metabolites levels in some cases, they do not mimic the natural delivery of gut microbial–derived small molecules through the portal circulation to the liver. To our knowledge, we describe here a novel surgical method to continuously deliver precise amounts of gut microbial metabolites intraportally to better recapitulate the natural systemic delivery route of microbial metabolites to the liver. This model will improve the interrogation of gut microbial metabolites and their associations to disease by providing an unmatched level of control when manipulating the portal blood metabolome.

Gut microbes are key modulators of human physiology and are appreciated as primary producers of metabolites that have been shown to impact human health (2). Disease states, including cardiovascular disease, chronic kidney disease, diabetes, cancer, and cancer therapeutic efficacy, all contain a demonstrable microbial metabolite component (3–7). However, most mechanisms by which gut microbial metabolites impact the host are poorly understood. Furthermore, a limiting factor in the mechanistic interrogation of microbial metabolites is the lack of physiologically relevant delivery mechanisms.

The canonical route of absorption for molecules originating from the gastrointestinal tract is venous drainage via the portal vein to the liver. This route of absorption is often crucial by allowing the liver to biotransform these molecules prior to entering the peripheral circulation. In many cases, molecules delivered via the portal vein to the liver are subjected to extensive presystemic hepatic metabolism, also known as the liver first-pass effect, such that their biotransformed products are the predominant forms seen in the peripheral circulation (8). It is appreciated that the portal blood metabolome can impact hepatic physiology in the context of numerous diseases, such as alcoholic liver disease, nonalcoholic fatty liver disease, and hepatocellular carcinoma (9, 10). As such, the administration route of gut-derived metabolites becomes critical, in which delivery of any particular metabolite should be done in a manner that recapitulates the natural in vivo delivery. Currently, common routes of administration include i.v., i.p., and s.c. injections, through intermittent oral gavage, or by incorporation into the diet or drinking water. Critically, none of these approaches ensure that the metabolite of interest remains unmodified by intestinal or gut microbial metabolism as it passes through portal circulation to the liver, in order to mimic normal physiologic conditions.

The dietary nutrients choline, phosphatidylcholine, and carnitine can serve as precursors for bacterial trimethylamine (TMA) generation, which is subject to further hepatic oxidation by flavin monooxygenases (3, 11). This 2-step meta-organismal metabolic process yields the proinflammatory, proatherosclerotic, and prothrombotic metabolite trimethylamine-*N*-oxide (TMAO) (3, 11–13). Owing to the well-studied nature of the TMA/TMAO pathway and the robust characterization of murine plasma TMA and TMAO pools, we selected TMA as a candidate molecule for a proof-of-concept study for our novel surgical procedure for continuous intraportal infusion. Additionally, TMA has a relatively low rate of first-pass hepatic metabolism, which is evident from the ability to manipulate peripheral plasma TMA through incorporation of its dietary precursors into rodent chow (11). Consequently, TMA presented itself as an ideal candidate molecule for a preliminary proof-of-concept study, given its ability to be detected in peripheral plasma resulting from low first-pass hepatic metabolism.

Gut microbial metabolism of dietary input yields a portal blood metabolome rich in microbe-derived metabolites that are delivered to the liver via portal circulation (9, 14). Polyphenols are abundant in fruits and vegetables and undergo extensive microbial metabolism (14, 15). Although polyphenolic flavonoids alone have demonstrated efficacy in the context of various inflammatory disorders (16–18), flavonoid-mediated attenuation of disease in disorders such as arthritis, obesity, and atherosclerosis has been reported to be microbe dependent (19–21). The dietary flavonol kaempferol has been described to undergo specific bacterial cleavage of its C-ring to yield a microbial flavonoid metabolite, 4-hydroxyphenylacetic acid (4-HPAA) (22, 23). Of clinical relevance, Hoyles and colleagues previously reported 4-HPAA negatively correlated with fat-free mass, waist circumference, and AUC for an oral glucose tolerance test in a study of 105 obese females originating from Spain and Italy (24). A key characteristic of 4-HPAA that warranted its inclusion in this study is that it undergoes rapid elimination following i.v. injection (25). To study a metabolite with such a short half-life, continuous delivery is required to more fully recapitulate in vivo conditions.

Here, we describe a surgical approach to continuously administer microbial and other gut-derived metabolites intraportally. We hypothesized that this technique would allow interrogation of the impact that microbial metabolites have on host physiology when delivered in a pharmacokinetically appropriate setting. Our findings suggest that the continuous intraportal infusion of TMA is sufficient to substantially raise plasma levels of both TMA and its primary hepatic metabolite TMAO in mice when compared with single-housed, sham, and saline control groups. Moreover, we demonstrate that continuous infusion of a metabolite with high first-pass metabolism, 4-HPAA, accumulates in the liver, whereas both the free and total plasma concentrations remain unchanged. Additionally, the continuous infusion of 4-HPAA is sufficient to alter the hepatic transcriptional landscape when compared with the vehicle control group.

## Results

### A surgical procedure for the continuous intraportal infusion of microbial metabolites.

**Figure 1:**
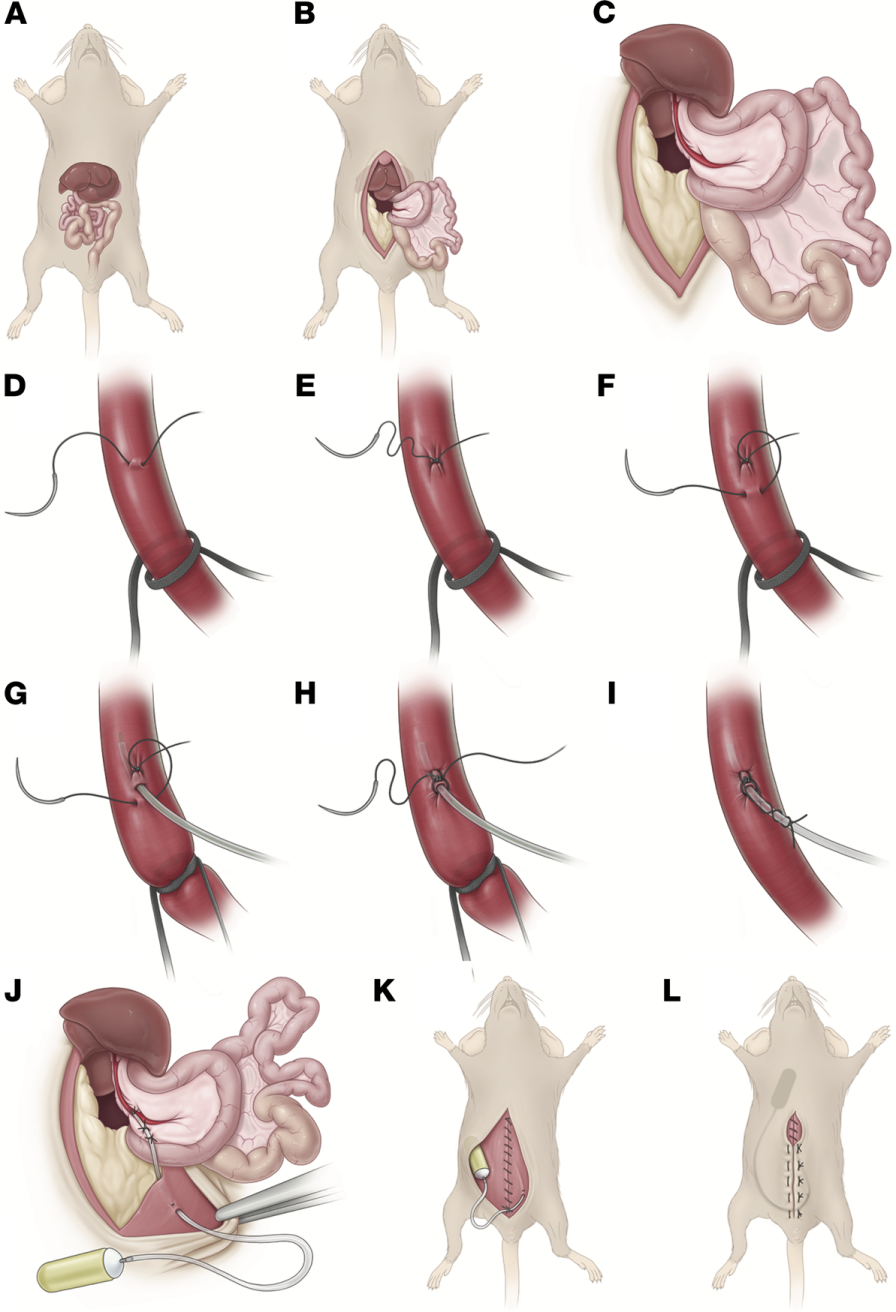
Stepwise illustration of portal vein cannulation procedure. (**A**) Abdominal anatomy. (**B**) Midline incision and laparotomy, with leftward externalization of intestines and overlying duodenum. (**C**) Externalization of left liver lobe superiorly. (**D–F**) Proximal vessel loop is loose. Using a 10-0 nylon micro suture, a stitch is placed in the anterior one-fourth of the vein, 3 mm distal to the vessel loop. The needle from this stitch is placed in the anterior one-fourth of the vein 1 mm proximal to the stitch. (**G**) The vessel loop is pulled taught. The guide wire is used to facilitate insertion. (**H**) The guide wire is withdrawn. The suture ends are tied together, slightly crimping the catheter. (**I**) The vessel loop is loosened and cut. Each of the tails of the suture are wrapped around the catheter twice and tied to create a “Chinese finger trap” effect. (**J**) Two anchor stitches are placed in the mesentery of the duodenum to secure the catheter. The catheter is externalized from the left lower quadrant and anchored to the peritoneal side of the abdominal wall. (**K**) The abdominal wall is closed, and the catheter anchored to the inferior aspect of the closure. (**L**) The osmotic pump is placed in the interscapular s.c. space. The skin is closed. Reproduced with permission from the Cleveland Clinic Center for Medical Art & Photography ©2021. All Rights Reserved.

The surgical procedure in its entirety is stepwise illustrated in Figure 1 and is described in detail in Methods. It is critical to ensure that strict adherence to sterile technique is maintained for the duration of the procedure given this is a survival surgery designed for long-term metabolite infusion. First, a laparotomy is performed, the intestines are externalized, the duodenum is rotated (Figure 1B), and then the left liver lobe is externalized to expose the portal vein (Figure 1C) (Supplemental Video 1; supplemental material available online with this article; https://doi.org/10.1172/jci.insight.145607DS1). Next, a polyurethane catheter is advanced within the portal vein using a Teflon-coated guide wire to facilitate entry and secured to the portal vein with a microsuture (Figure 1, D–I, and Supplemental Video 2). The catheter is then externalized from the peritoneal cavity, where it is connected to a mini osmotic pump (Figure 1J and Supplemental Video 3). The osmotic pump is placed within the s.c. tissue of the interscapular space on the dorsum of the mouse, and the abdominal wall and skin are closed (Figure 1, K and L).

**Figure 2:**
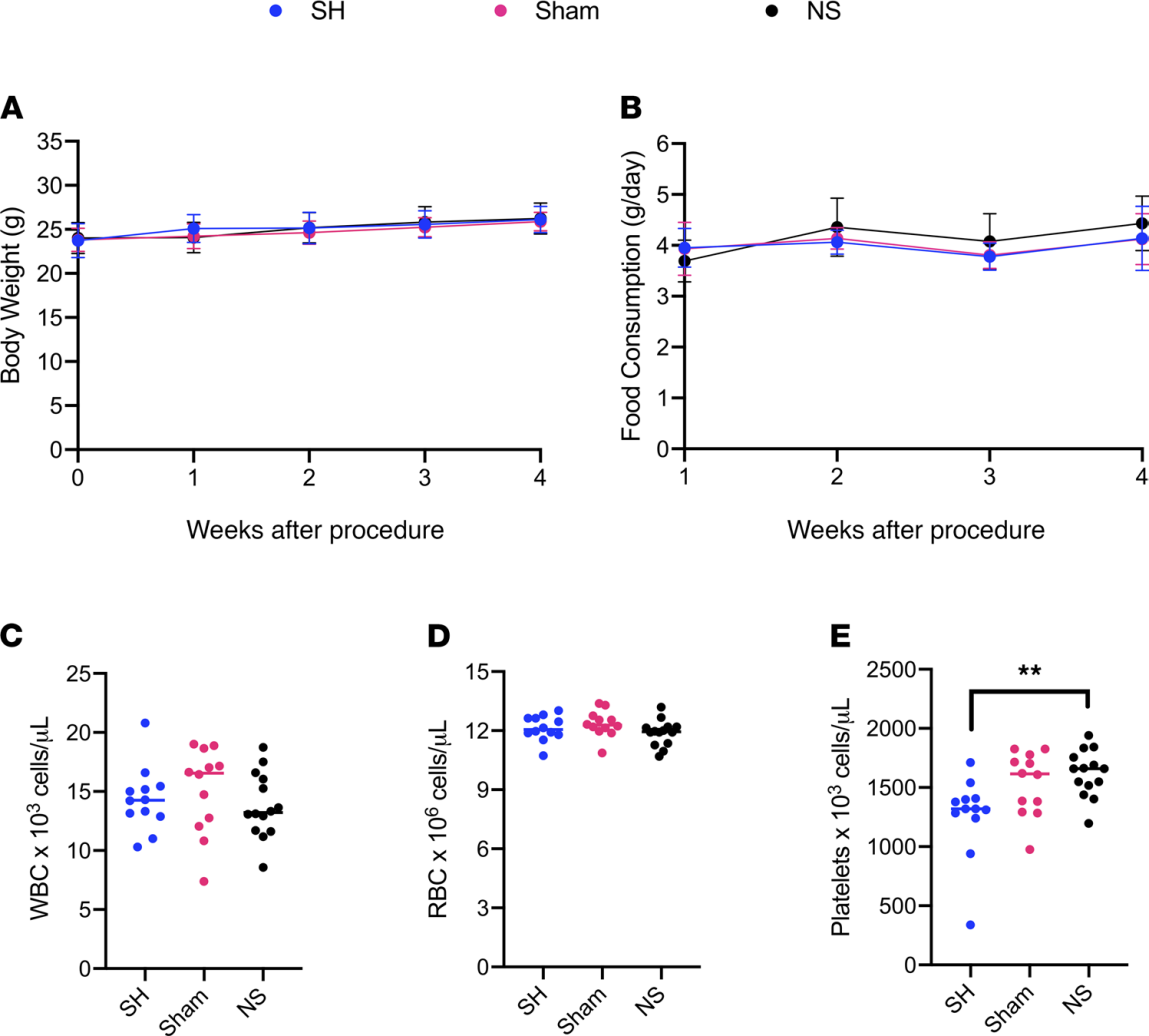
Health status monitoring of mice undergoing portal vein cannulation. Portal vein cannulation was performed using saline-filled osmotic pumps (NS) and compared with sham procedure (Sham) and single-housed (SH) controls (*n* = 12–14 per group). (**A**) Body weights and (**B**) food consumption were measured weekly. (**C**) WBC, (**D**) RBC, and (**E**) platelet counts were measured 1 week after surgery. Statistical analysis of **A** and **B** was performed using a 1-way ANOVA with Tukey’s multiple comparisons test on the AUC and data are shown as the mean ± SEM. Statistical analysis of **C–E** was performed with 1-way ANOVA with Tukey’s multiple comparisons test. **P < 0.01.

Portal vein cannulation was performed using saline-filled osmotic pumps and compared with sham procedure and single-housed controls to investigate its physiologic impact. No changes in body weight (Figure 2A) or food consumption (Figure 2B) were observed over 4 weeks. One week after surgery, no difference was seen in WBC or RBC counts (Figure 2, C and D), although platelet counts were found to be higher when compared with single-housed mice but not sham controls (Figure 2E). Moreover, 1 week after surgery, no observable differences in energy expenditure, ambulatory activity, or locomotor activity were observed (Supplemental Figure 1). Taken together, these data suggest the surgery is well tolerated, underscoring the resilience of *Mus* *musculus* as a model system for surgical procedures such as the one described here.

**Figure 3:**
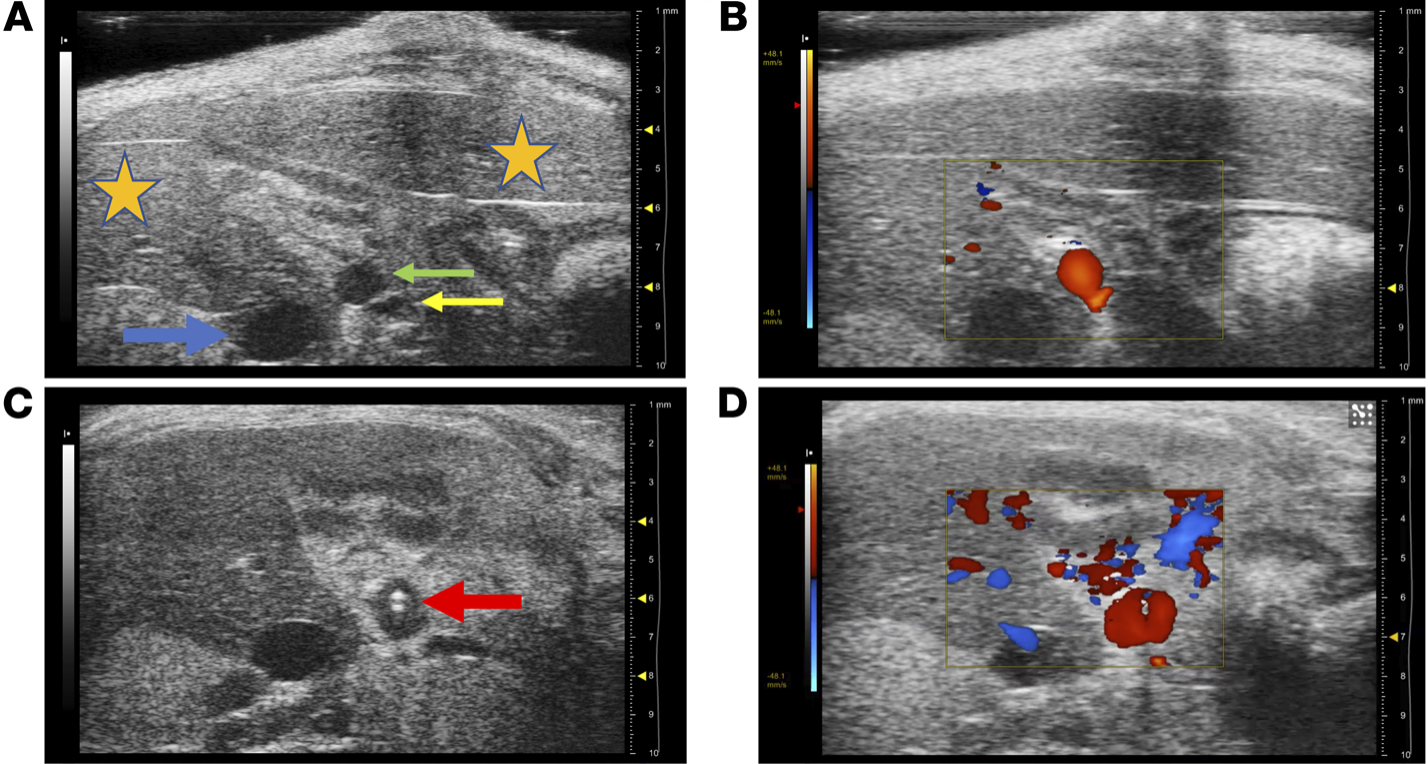
Ultrasound imaging of catheterized portal vein. Mice underwent ultrasound imaging 1 week after surgery (*n* = 3). (**A**) The portal vein proximal to the catheter appeared patent and nondilated, and (**B**) demonstrated liver-directed laminar flow on Doppler imaging. (**C**) The tip of the catheter was visualized within the distal portal vein (red arrow), and (**D**) liver-directed laminar flow was visualized around the catheter. (Orange star, liver; green arrow, portal vein; yellow arrow, hepatic artery; and blue arrow, inferior vena cava.)

Ultrasound imaging demonstrated the tip of the cannula properly positioned within the portal vein and surrounding liver-directed laminar blood flow 1 week after surgery (Figure 3) (Supplemental Videos 4 and 5). At the completion of the experiment, mice were placed under isoflurane anesthesia, and saline was gently injected through the cannula to ensure catheter patency and position (Supplemental Video 6).

### Continuous infusion of TMA provides proof of concept for the surgical procedure.

**Figure 4:**
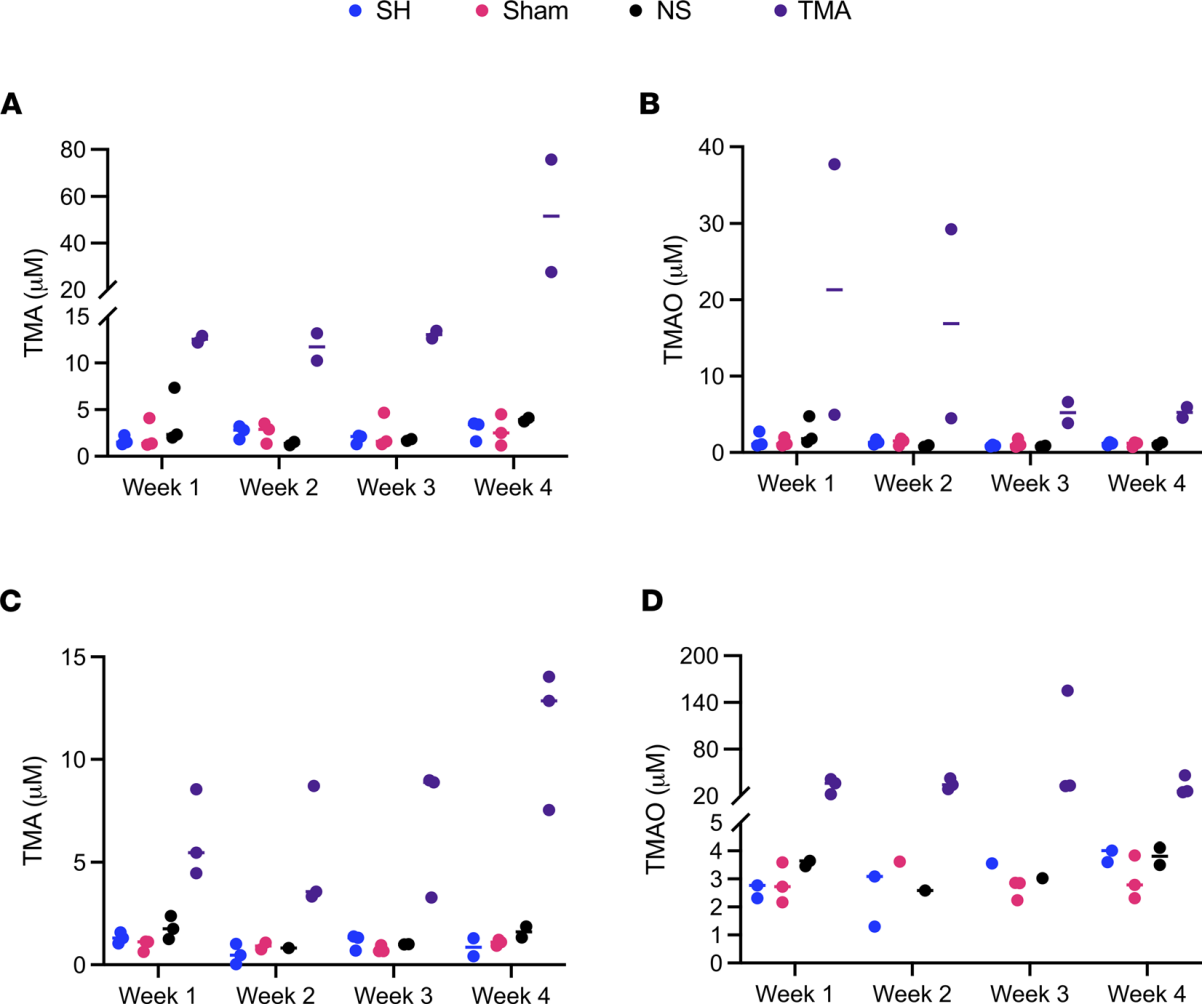
Continuous infusion of TMA — a microbial metabolite with low hepatic first-pass metabolism. (**A**) Peripheral plasma levels of trimethylamine (TMA) in male C57BL/6 mice receiving TMA via intraportal catheter (*n* =2–3). Single-housed controls (SH), sham surgery controls (Sham), and osmotic pump normal saline controls (NS) are also shown. (**B**) Peripheral plasma trimethylamine-*N*-oxide (TMAO) levels in male C57BL/6 mice. (**C**) Peripheral plasma levels of TMA in female C57BL/6 mice (*n* = 2-3). SH, Sham, and NS controls are also shown. (**D**) Peripheral plasma TMAO levels in female C57BL/6 mice.

As a preliminary proof of concept, we administered TMA continuously for 4 weeks in both male (Figure 4, A and B) and female (Figure 4, C and D) C57BL/6 mice. As recovery after surgery requires each mouse to be single-housed, we included a single-housed control group (SH) when measuring plasma TMA and TMAO. Additionally, we included sham procedure control animals that were opened via midline laparotomy and had their portal vein dissected but not cannulated (Sham). We also included control animals that had their portal vein cannulated and received normal saline from the osmotic pump for the duration of the study (NS). As expected, the continuous intraportal infusion of TMA over 4 weeks led to a marked increase in peripheral plasma TMA levels in male mice when compared with saline control animals as measured by liquid chromatography with on-line tandem mass spectrometry (LC-MS/MS) (Figure 4A), although peripheral plasma TMAO was only slightly elevated (Figure 4B). Conversely, female mice had only slightly higher peripheral plasma concentration of TMA (Figure 4C) with markedly higher peripheral plasma levels of the host liver-derived cometabolite TMAO (Figure 4D). These data are further corroborated by previous studies demonstrating that female mice have a more than 1000-fold higher expression of flavin monooxygenase 3 (FMO3), the main enzyme in the liver responsible for the conversion of TMA to TMAO (3, 26). Hence, the higher TMA/TMAO ratio in males and concomitantly lower TMA/TMAO ratio in females was expected. Collectively, these data provide proof of concept that the surgical model of continuous intraportal infusion of microbial metabolites is feasible.

### Continuous infusion of 4-HPAA — a microbe-derived metabolite with high first-pass hepatic metabolism.

**Figure 5:**
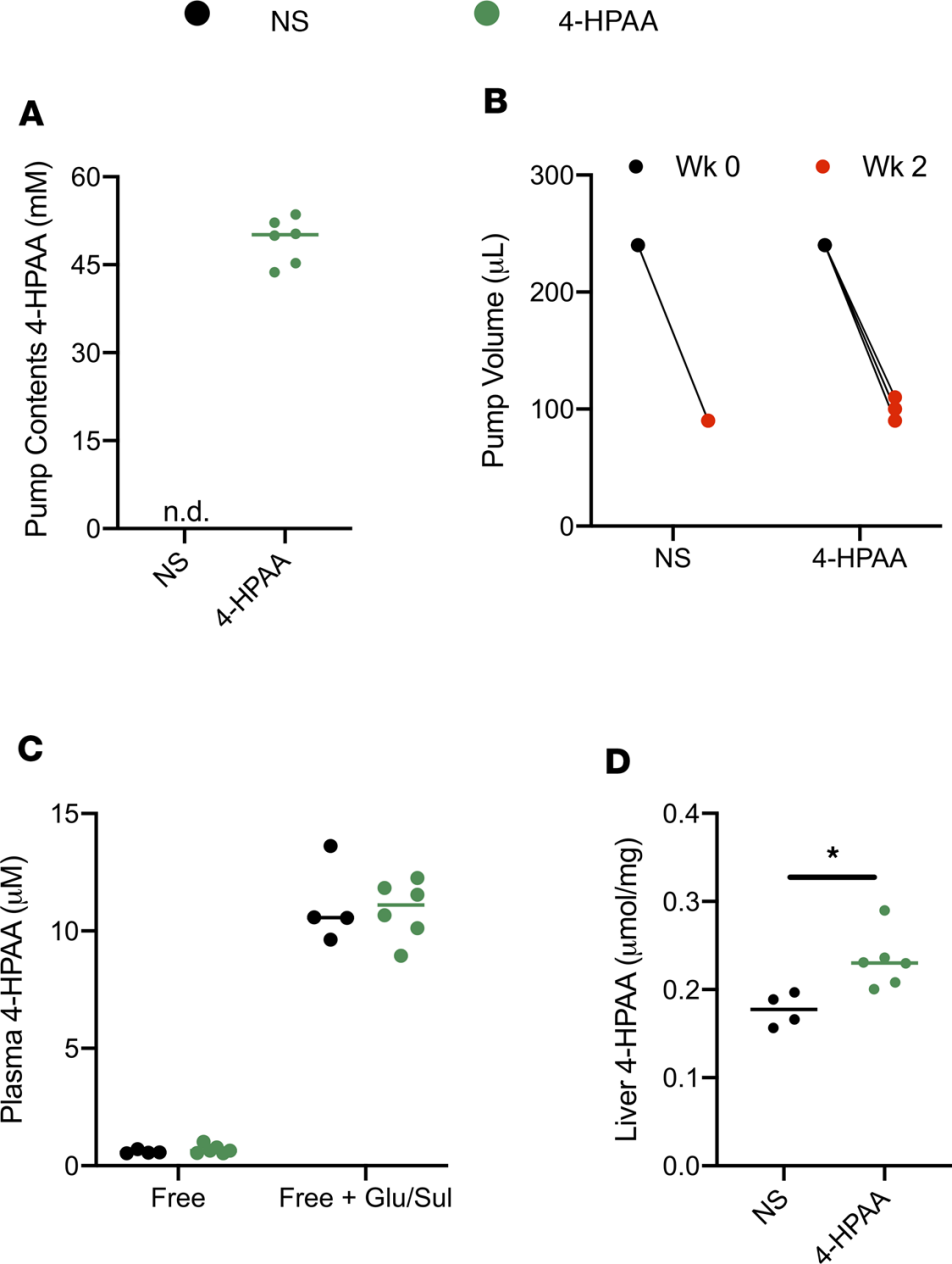
Continuous infusion of 4-HPAA — a microbial metabolite with high first-pass metabolism. (**A**) Osmotic pumps were filled with either normal saline (NS) or 4-hydroxyphenylacetic acid (4-HPAA). (**B**) Volume of either normal saline or 4-HPAA dispensed from the osmotic pumps after 2 weeks of continuous intraportal infusion (*n* = 4–6). (**C**) A larger proportion of the 4-HPAA pool circulates in a glucuronidated/sulfated (Glu/Sul) form, reaching 10-fold higher total peripheral plasma 4-HPAA concentrations compared with the free 4-HPAA pool. (**D**) Significantly more 4-HPAA is detected in the liver (free + Glu/Sul), the last anatomic location where differential abundance of 4-HPAA is observed resulting from high first-pass hepatic metabolism of 4-HPAA. *P* values were calculated using an unpaired 2-tailed *t* test. *P < 0.05.

Next, we sought to determine the efficacy of our surgical model using a metabolite with a rapid rate of hepatic clearance, 4-HPAA. Osmotic pumps with either normal saline or 4-HPAA dissolved in normal saline (Figure 5A) were implanted following the surgical procedure previously outlined and were allowed to continuously infuse their contents for 2 weeks (Figure 5B). As expected, owing to the previously reported rapid clearance of 4-HPAA (25), we did not observe any difference in either free or glucuronidated/sulfated (Glu/Sul) levels of 4-HPAA in peripheral plasma between normal saline and 4-HPAA groups (Figure 5C). Moreover, we gained insight into the overall circulating pool of 4-HPAA, where it was observed that 4-HPAA in plasma is either Glu/Sul as measured by LC-MS/MS (Figure 5C).

The liver is uniquely positioned to act as a central player in metabolite-mediated gut-liver crosstalk and ultimately controls the fate of many gut-derived metabolites. In support of this concept, a significant accumulation of free and Glu/Sul 4-HPAA was observed in the livers of mice receiving 4-HPAA compared with control mice (Figure 5D). In summary, these data suggest that much of the circulating 4-HPAA pool is Glu/Sul, plasma levels of 4-HPAA cannot be raised owing to rapid hepatic clearance, and that accumulation of 4-HPAA occurs in the liver after continuous intraportal infusion.

To determine the effect of 4-HPAA on biomarkers of hepatic injury, plasma aspartate transaminase (AST) and alanine transaminase (ALT) were measured after 2 weeks of intraportal delivery of either saline or 4-HPAA and compared with single-housed mice (Supplemental Figure 2). Intraportal cannulation itself does not increase AST or ALT levels when compared with a single-housed mouse. Consistent with previous studies highlighting 4-HPAA as a hepatoprotective molecule, ALT was significantly reduced in 4-HPAA–treated mice when compared with single-housed mice (Supplemental Figure 2B) (27).

### Physiologic administration of 4-HPAA into the portal vein alters the transcriptional landscape of the liver.

**Figure 6:**
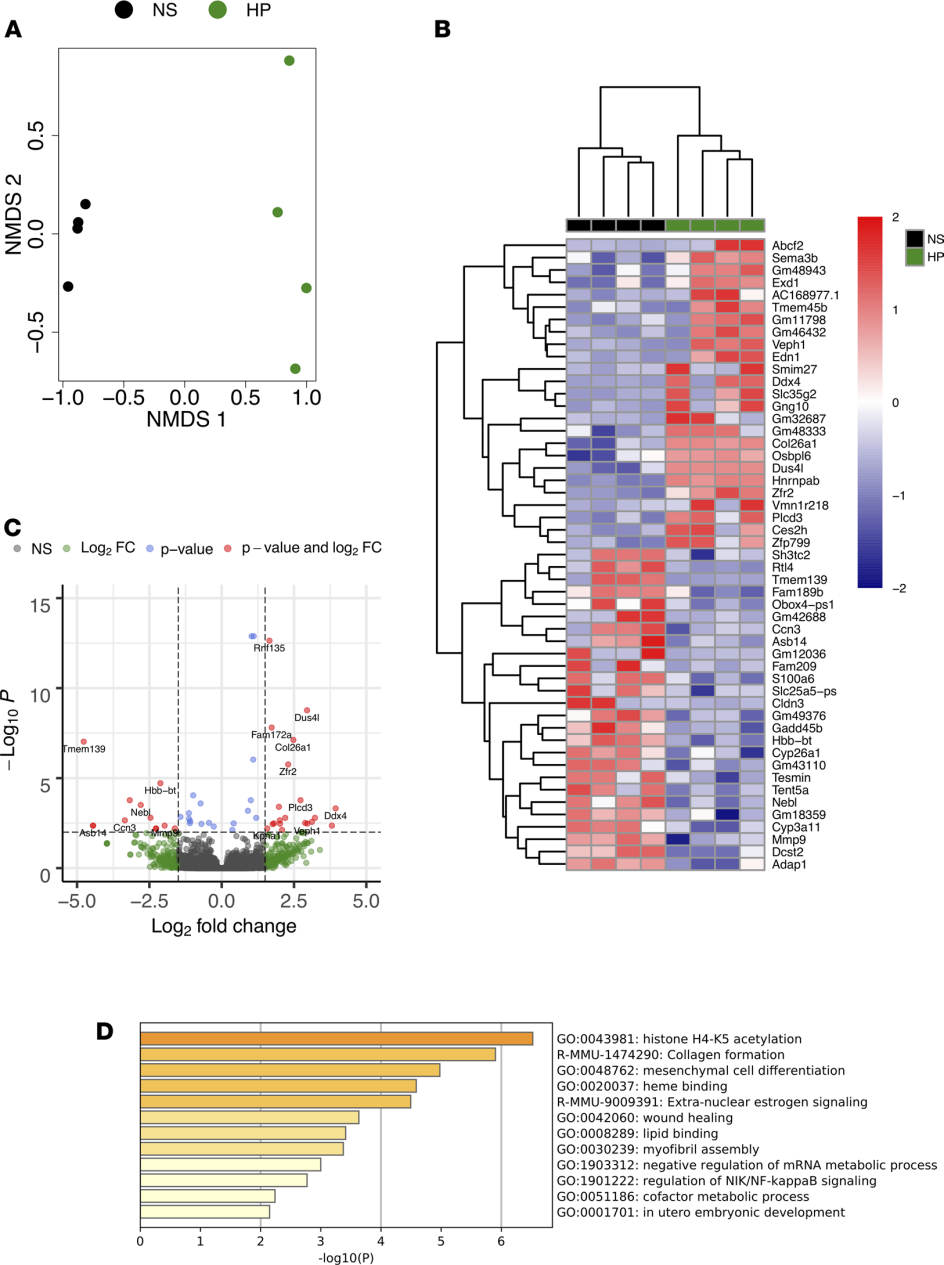
Continuous portal infusion of 4-HPAA changes the hepatic transcriptional landscape. (**A**) Nonmetric multidimensional scaling (NMDS) of RNA-Seq transcriptome data representing the hepatic gene expression signature of the top 500 differentially expressed transcripts between 4-HPAA–treated mice (green) relative to normal saline control mice (black) as sorted by log_2_ fold change. NMDS was performed using DESeq2 normalized counts. (**B**) Heatmap of hierarchically clustered differentially expressed genes arranged by adjusted *P* value and log_2_ fold change. The *z* score normalized values scaled by row. (**C**) Volcano plot of RNA-Seq transcriptome data representing hepatic gene expression signature of 4-HPAA–treated mice relative to normal saline control mice. Genes highlighted in red correspond to those that are significantly differentially expressed (adjusted *P <* 0.001) with a log_2_ fold change > 1.5. (**D**) Gene ontology assignments of the top 100 differentially expressed genes as sorted by adjusted *P* value. *n* = 4 per group for all RNA-Seq analyses.

Since 4-HPAA administration resulted in increased levels in the liver, we studied the local effects of the compound. We performed unbiased RNA sequencing in liver tissues from mice receiving continuous infusion of either saline or 4-HPAA (Figure 6). Nonmetric multidimensional scaling (NMDS) and hierarchical clustering showed clear separation and predictable assignment of both saline and 4-HPAA groups (Figure 6, A and B). The continuous infusion of this single microbial metabolite caused marked global hepatic transcriptional changes when compared with the normal saline control (Figure 6C). In total, 116 genes were differentially expressed between normal saline control and 4-HPAA–treated mice (Supplemental Data File 1). 4-HPAA has been previously reported to mediate lipid metabolism and the resolution of inflammation (27, 28). Consistent with these findings, our hepatic RNA sequencing data implicate 4-HPAA in the regulation of lipid metabolism and NF-κB signaling (Figure 6D). Taken together, these data suggest that the administration of a single microbial metabolite, 4-HPAA, is sufficient to alter the hepatic transcriptional landscape, providing the first clues into how the gut microbe–derived metabolite 4-HPAA can impact host liver gene expression.

## Discussion

Attempts to achieve long-term access to the portal circulation for continuous infusion or interval bolus administration have received attention over the last few decades. Although mice are the most commonly used vertebrate in research, there are few examples of long-term administration of compounds into the portal circulation given the small size and fragility of the mouse portal vein. Rather, most portal vein cannulations are performed in larger rat and porcine models (29, 30). To our knowledge, our surgical technique is the first to use an indwelling pump along with portal vein cannulation to achieve continuous infusion over extended time periods. Additionally, the only 2 papers to describe portal vein continuous infusion in mice use an external swivel wheel and syringe pump with 5-foot-long catheter tubing and infusion rate of 6 mL/day (1000-fold higher than our model) (31, 32). That model, along with all others describing portal vein cannulation for interval bolus administration, does not perform a confirmation procedure at necropsy to ensure proper intraportal position of the catheter tip (33–35). This is crucial as slight pressure on the sidewall of the portal vein will lead to erosion of the catheter into the vein sidewall or adjacent tissues. Additionally, all other models employ the i.p. use of surgical glue at the catheter — portal vein insertion side, although glue is often nonsterile, produces an inflammatory reaction with dense adhesion formation, and affects gut peristalsis if accidentally applied on bowel (36). However, the use of surgical glue in this model may prevent catheter dislodgement over time and be a helpful adjunct to ensure proper maintenance of intraportal cannula position for long-term studies.

We believe the method described here of portal vein cannulation and continuous infusion is novel in its ability to achieve precise administration of microbial metabolites in a physiologically relevant way. Although the discussion of pharmacokinetic experimental design aimed at liver metabolism is outside the scope of this discussion, a necessary precursory step of this model involves understanding a metabolite’s rate of hepatic first-pass metabolism, volume of distribution, half-life, clearance and elimination, peripheral plasma concentration, portal plasma concentration, and possibly liver tissue concentration in order to assess its suitability for this model and to properly calculate the necessary infusion to achieve a desired concentration change. Achieving long-term patency and the proper intraportal position of the catheter is technically challenging, and it is the hope of the authors that the detail provided here will allow its more widespread use. The extensive s.c. dissection, length of surgery (approximately 50 minutes), and need to single-house animals should increase the thoughtfulness paid to a metabolite’s suitability for this technique (e.g., rapid hepatic first-pass metabolism and involvement in enterohepatic circulation). These confounding factors also support the role of this method as an adjunct to traditional methods of drug administration, such as oral feeding or gavage, i.p. injection or infusion, and i.v. injection or infusion when possible.

The method described here provides the ability to study microbial metabolite-mediated gut-liver crosstalk, with an unparalleled level of resolution in a physiologically relevant manner. Two microbial metabolites were administered to illustrate the model’s utility and to highlight the importance of liver first-pass metabolism when examining microbial metabolite connections to host physiology and disease. Hepatic first-pass metabolism (also known as liver first-pass effect) refers to the degree to which a molecule delivered to the liver via the portal vein is transformed prior to entrance into the systemic circulation (8). The higher a molecule’s first-pass metabolism, the lower peripheral extrahepatic tissue exposure. Hence, administration of gut-derived microbial metabolites with high first-pass metabolism benefit from portal vein delivery as peripheral (e.g., i.v., and s.c.) administration is nonphysiologic. Trimethylamine is a well-characterized microbial metabolite and of strong interest in our laboratory (13, 37–40). TMA’s conversion to TMAO is predominantly catalyzed by hepatic FMO3, and, therefore, undergoes a degree of liver first-pass metabolism. Pharmacokinetic studies investigating TMA metabolism performed in male Wistar rats observed a 2-hour half-life and relatively low liver first-pass metabolism, demonstrating most of the TMA within the portal vein is delivered to the systemic circulation (41, 42). As such, portal vein delivery of TMA increases peripheral plasma concentrations, making it well suited for a proof-of-concept experiment. We observed appreciable rises in both TMA and TMAO peripheral plasma concentrations when administered via portal vein to 8-week-old C57BL/6 male and female mice (Figure 4). It is also important to note that plasma TMA and TMAO levels were sexually dimorphic, which is in agreement with previous reports (3, 26).

The metabolite 4-HPAA is one of several phenolic acids generated from gut microbial polyphenol and amino acid metabolism (22, 23). Preliminary studies demonstrated its relatively high hepatic first-pass effect (data not shown), with an appreciable proportion undergoing glucuronidation or sulfation. As such, 4-HPAA would greatly benefit from portal vein over peripheral administration. As expected, 4-HPAA administration did not produce differences in peripheral-free or Glu/Sul fractions of 4-HPAA compared with normal saline vehicle controls (Figure 5C). However, liver levels of total 4-HPAA were increased (Figure 5D). These results highlight the importance of direct intraportal delivery to achieve a physiologically relevant model.

The study of gut microbes in human health and disease is rapidly expanding. Portal vein delivery and hepatic first-pass metabolism is a fundamental aspect of many gut microbe–derived metabolites. This method will allow for interrogation of gut microbial metabolites and their links to disease in genetically tractable mouse models. Leveraging this portal vein infusion approach in mice genetically lacking suspected host receptors will be a powerful manner to identify new microbe host signaling pathways relevant to disease pathogenesis (43). A special related application of this method is the interrogation of bacterial and host lipid signaling given their rapid metabolism when delivered from the gut to liver, making i.p. and other forms of systemic administration less relevant. Other pharmacokinetic and xenobiotic metabolism beyond only microbe-derived metabolite studies will also benefit from applications of this technique. The enterohepatic circulation involves a unique niche of portal vein-enriched metabolites often mediating gut-liver crosstalk to generate vast and largely understudied physiologic effects. This surgical approach will be especially useful in selectively modifying metabolites in this portal blood niche to examine the long-term changes in mouse models of disease.

## Methods

Note that strict adherence to sterile technique is crucial, as contamination may compromise pump and catheter contents.

### Specialized materials.

Specialized materials include a Leica Wild M650 surgical microscope (operation was performed under ×6 to ×25 magnification); a tabletop rodent anesthesia machine with chamber and nose cone (Parkland Scientific, V3000PK); a rat intrathecal catheter (short) (Alzet, 0007741; distal most flexible portion is 0.36 mm outer diameter [OD], 0.18 mm inner diameter [ID]; middle less flexible portion is 0.84 mm OD, 0.36 mm ID; proximal least flexible portion is 1.02 OD, 0.61 mm ID) (the catheter includes a Teflon-coated stylet/guide wire; note that smaller diameter catheters led to clot formation within the catheter tip lumen); polyethylene tubing (Alzet, 0007750); an osmotic pump (Alzet, 2004, with a rate of infusion 0.25 μL/h, up to 4 weeks duration); an infrared warming pad (Kent Scientific, DCT-15); 10-0 nylon micro suture (AroSurgical, T04A10N07-13); 4.75 x 4 inch transparent film dressing (3M Tegaderm, 1626W); and small drape with adhesive aperture (3M, 1092).

### Filling and priming of osmotic pump.

The procedure used for filling and priming of the osmotic pump is as follows. A sterile drape was laid within a laminar flow hood. The osmotic pump, polyethylene tubing, and filler needle (provided by Alzet) were placed on the drape, along with a 1 mL syringe. A vial of solution was opened. Sterile gloves were donned. The flow modulator portion of the osmotic pump was removed. Using the syringe and filler needle (ensure no air bubbles are within the needle or syringe), the osmotic pump was slowly filled, and the precise amount of the solution documented. The flow modulator was reinserted. The polyethylene tubing was attached (not the catheter) to the pump. The pump with attached tubing was placed in a small saline-filled beaker, ensuring the pump was completely immersed. The tubing was draped over the side of the beaker, and then the beaker was covered with aluminum foil and placed in a 37°C incubator for 40 hours (specific to pump model 2004).

### Surgical method.

Surgical procedures were performed as follows. (Step 1) Induction of anesthesia was achieved with 3%–4% isoflurane. (Step 2) The abdomen and flank of the mouse were shaved, and then sterile eye ointment was applied. (Step 3) The mouse was placed on an infrared warming pad. Anesthesia was maintained with 2%–3% isoflurane administered via nose cone. The mouse was secured to the warming pad with silk tape. Note that the upper extremities should be taped anterior on the nose cone to allow room for later placement of the osmotic pump in the interscapular space. (Step 4) The abdomen was sterilized using 10% betadine and 70% ethanol. Buprenorphine 0.1 mg/kg was administered s.c. Bupivacaine 6 mg/kg was administered s.c. along the planned incision. (Step 5) A sterile transparent adhesive film and procedure drape were applied (see *Specialized materials*). (Step 6) The catheter and instruments were placed on the field. The catheter was prepared by cutting the distal, smallest diameter portion to about 2.5 cm, or one-third the length of the mouse. The guide wire was then cut to be slightly longer than the catheter and placed within the catheter lumen to protrude about 1 mm from its distal tip. (See Supplemental Video 1 for the following 5 steps.)

(Step 7) We next made a midline vertical skin incision, and the surrounding s.c. plane was dissected to aid with exposure. (Step 8) The s.c. plane of the right lower quadrant was dissected free. A curved hemostat was then used to dissect the s.c. plane from the right lower quadrant to the interscapular space on the dorsal side of the mouse; minimal resistance should be encountered. Then, a stitch using 6-0 silk suture was placed within the dermis of the right flank, and its tails were left long to be used as a future catheter anchor stitch. (Step 9) A laparotomy was performed from the suprapubic region to the xiphoid process, dissecting the xiphoid free of its lateral attachments. (Step 10) The intestines were externalized leftward and wrapped in moistened gauze (Figure 1B). Note that prevention of twisting of the mesentery is essential to maintain intestinal arterial and venous flow. A stitch using a 6-0 silk suture was placed on the peritoneal/interior side of the left lower quadrant abdominal wall, and its tails were left long to be used as a future catheter anchor stitch. (Step 11) The duodenum was rotated laterally to expose the portal vein (Figure 1B). The left lobe of the liver was externalized superiorly over the xiphoid process and held in place with moistened gauze to expose the liver hilum (Figure 1C). Note that a moistened gauze and the pressure of a curved hemostat on the duodenum may aid in maintaining exposure. Additionally, a 4-hour fast (withholding diet but not water) prior to surgery may decrease gastric and bowel distention and aid exposure. (See Supplemental Video 2 for the following 8 steps.)

(Step 12) The portal vein was then carefully dissected near the splenic vein–superior mesenteric vein confluence. A vessel loop was placed just distal to the confluence using 6-0 silk suture and left loose. Note that care should be taken to minimize violation of the pancreas. (Step 13) Approximately 3 mm distal to the vessel loop, a stitch was placed in the anterior one-fourth of the portal vein using a 10-0 nylon micro suture (Figure 1, D and E). The suture tails and needle were not cut. Note that the distal portal vein may be gently compressed with forceps to dilate the vein proximally and assist with placement of this stitch. (Step 14) The needle of the prior step was then passed through the anterior one-fourth of the portal vein 1 mm proximal to the stitch placed in the previous step (Figure 1F). No knot was tied. (Step 15) The vessel loop was gently pulled inferiorly to occlude flow and produce slight tension on the vein. The catheter guide wire apparatus was inserted between the stitch and suture placed in the previous 2 steps (Figure 1G). Note that the point of insertion should be distal to the insertion of the splenic vein to reduce turbulent flow and the risk of vessel thrombosis. The catheter was advanced about 5 mm, and the guide wire was retracted about 1 cm. Note that the knot placed in the preceding step may be retracted anteriorly slightly to aid in catheter guide wire insertion. Minimal bleeding should occur around the catheter insertion site. (Step 16) The catheter was pulled back such that its tip protruded approximately 3 mm within the vein. Note that further insertion of the cannula increases the risk of clot formation and portal vein occlusion. The free ends of suture in the preceding steps were tied to secure the portal vein around the catheter with such force to slightly crimp the catheter (Figure 1H). The vessel loop was loosened and cut. Note that portal vein occlusion time should be under 5 minutes. Additionally, surgical glue may be used to secure the catheter in place of micro suture, although glue is often nonsterile, produces an inflammatory reaction with dense adhesion formation, and affects gut peristalsis if accidentally applied on bowel. However, the use of surgical glue may prevent catheter dislodgement over time and be a helpful adjunct to ensure proper maintenance of intraportal cannula position for long-term studies. (Step 17) The free tails of the suture from the preceding step were wrapped around the catheter twice and tied to create a “Chinese finger trap” effect (Figure 1I). (Step 18) Two stitches were placed to anchor the catheter to the mesentery of the duodenum (Figure 1J). The mesentery should be freed from any retraction, and no tension should be present on the catheter–portal vein junction; otherwise, the position of the mesentery anchor stitches should be adjusted. The catheter should take a path parallel to the natural trajectory of the portal vein, as pressure of the catheter against the sidewall of the portal vein will lead to erosion into the vein sidewall or adjacent tissues. Note that glue may be used in place of suture to anchor the catheter along the duodenal mesentery, although glue has the aforementioned disadvantages. (Step 19) Then, the guide wire was fully removed. A 22-gauge needle attached to a 1 mL syringe was placed in the lumen of the catheter. Blood was aspirated to remove air from the catheter. About 100 μL saline was then slowly infused to clear the catheter of blood, and the catheter was clamped at its proximal position. (See Supplemental Video 3 for the following 6 steps.)

(Step 20) The 22-gauge needle was used to pierce the external left lower quadrant of the abdominal wall (not including the skin) near the anchor stitch placed previously. The catheter was then pulled over the needle, and the needle-catheter was withdrawn to achieve externalization of the catheter from the left lower quadrant (Figure 1J). Note that the final orientation of the catheter should be such that no twists are present along its trajectory into the portal vein. The catheter was clamped at its proximal position, and the anchor stitch was then tied around the catheter to secure it to the abdominal wall. (Step 21) The duodenum, intestines, and then liver should be placed back into the abdomen in proper anatomic orientation. Final catheter patency and position were checked using the 22-gauge needle to gently aspirate portal blood and ensure smooth infusion of saline. The catheter was again clamped and attached to the osmotic pump. Note that slight retrograde portal blood flow into the distal one-third of the catheter was often observed and will not clot the catheter. (Step 22) Ketoprofen (5 mg/kg) was then administered into the peritoneal cavity, along with 0.5–1 mL saline to replace evaporative losses and any blood loss. (Step 23) The abdominal wall was closed with a simple continuous 6-0 polydioxanone suture, and the suture tails of the inferior aspect of the closure left long. The catheter then wrapped across midline and anchored using the suture tails from the inferior aspect of the abdominal wall closure (Figure 1K). (Step 24) The osmotic pump was placed in the interscapular s.c. space using the tunnel created previously (Figure 1, K and L), and secured with the anchor stitch placed previously. (Step 25) Next, the skin was closed in an interrupted horizontal-mattress fashion using a 5-0 polypropylene suture (Figure 1L).

### Postoperative monitoring and care.

Postoperative monitoring and care were performed as follows. The mouse was placed on a warming pad to recover and then single-housed to decrease chance of catheter displacement. Water and food were immediately offered, and moistened food pellets were placed on the cage floor. Buprenorphine 0.1 mg/kg was administered s.c. on the evening of surgery, and twice daily on postoperative days 1 and 2. Scruffing should be avoided to avoid worsening incisional pain, and displacement of the pump and catheter; rather, s.c. injection on the left dorsum of the mouse may be used for medication administration. Twice daily monitoring for 3 days and daily monitoring for 14 days are recommended. Special attention should be paid to pain status, activity level, hydration status, incision integrity, pump location, and passage of feces. Signs of distress such as severe pain and markedly decreased activity suggest portal vein occlusion, in which case immediate euthanasia should be performed. Sutures were removed after 14 days.

### Necropsy method.

(See Supplemental Video 6.) Induction and maintenance of anesthesia was achieved with 4% and 2% isoflurane, respectively. Adequate depth of anesthesia was confirmed by no response to toe pinch. Isoflurane anesthesia was maintained with a nose cone device, and the mouse was secured to a necropsy board. Both a laparotomy and thoracotomy were performed. Blood collection via cardiac puncture was performed. However, note that this should be performed prior to testing catheter placement and patency. The catheter was then dissected free and cut transversely near the inferior aspect of the previous abdominal wall closure. A 26-gauge needle attached to a 1 mL syringe of normal saline (devoid of air) was placed within the distal lumen of the freshly cut catheter. Saline was gently injected using forceps to compress the catheter tubing around the catheter. If the catheter is patent and its tip correctly within the portal vein, the liver parenchyma will blanch. The remainder of the necropsy and tissue collection was at the discretion of the surgeon, although the catheter tip location, portal vein, liver hilum, pancreas, and mesentery of the duodenum should be inspected for any abnormalities. The amount of solution within the osmotic pump should be documented and compared with the amount of solution originally used to fill the pump. Additionally, pump contents may be taken at this point for analysis to ensure integrity of the solution over the study period.

### Animals.

Eight- to ten-week-old male and female C57BL/6J mice were purchased from The Jackson Laboratory and housed under specific pathogen-free conditions at the Biological Resources Unit within the Cleveland Clinic Lerner Research Institute. Mice were given standard rodent chow and drinking water ad libitum. Induction and maintenance of anesthesia were achieved with 4% and 2% isoflurane, respectively. After surgery, mice were single-housed and given standard rodent chow and drinking water ad libitum. Perioperative mortality was less than 10%.

### Reagents.

Trimethylamine hydrochloride (MilliporeSigma; T72761) and 4-HPAA (Alfa Aesar; A15018) were dissolved in 0.9% normal saline (Baxter; 2F7124) at 9.87 M and 55 mM, respectively. These concentrations were based on flow rate and pharmacokinetic calculations aimed to raise plasma concentrations above the normal physiologic level. Before loading into the osmotic pump, each metabolite suspension was sterilized using a 22 μm pore vacuum filtration system.

### Oxymax/Comprehensive Animal Monitoring System metabolic cage studies.

For indirect calorimetry studies and activity monitoring, mice (*n* = 12) were single-housed in the Oxymax/Comprehensive Animal Monitoring System metabolic cage monitoring system (Columbus Instruments). The animals were allowed to acclimate for 24 hours at room temperature (23°C) before 48 hours of continuous data monitoring. Energy expenditure, ambulatory activity, and locomotor activity data were recorded.

### Ultrasound.

A Vevo 2100 (Visual Sonics) ultrasound was used for ultrasound imaging. Induction and maintenance of anesthesia was achieved with 4% and 2% isoflurane, respectively. After placement of the mouse on a warmed platform, hair removal cream was applied and gently removed with a paper towel and liberal application of sterile water. Ultrasound gel was applied, and an MS550S transducer used to image the portal vein and catheter within. Doppler imaging may be used to determine blood flow directionality, as well as turbulence around the catheter. After imaging was complete, the ultrasound gel was removed, and the mouse placed in a cage on top of a warming pad until awake and recovered.

### Quantification of TMA and TMAO in acidified plasma.

Stable isotope dilution high-performance LC-MS/MS was used for quantification of TMA and TMAO levels as previously described (3, 44). Their d9(methyl)-isotopologues, d9-TMA and d9-TMAO, were spiked into plasma as internal standards. LC-MS/MS analyses were performed on a Shimadzu 8050 triple quadrupole mass spectrometer. TMA, TMAO, d9-TMA, and d9-TMAO were monitored using multiple reaction monitoring of precursor and characteristic product ions as follows: *m/z* 60.2 **→** 44.2 for TMA; *m/z* 69.0 **→** 49.1 for d9-TMA; *m/z* 76.0 **→** 58.1 for TMAO; and *m/z* 85.0 **→** 66.2 for d9-TMAO.

### Sample preparation and quantification of 4-HPAA.

To deconjugate plasma samples, 20 μL was treated with β-glucuronidase/arylsulfatase obtained from *Helix pomatia* according to the manufacturer’s recommendation (Roche; 10127698001). Liver was extracted by homogenizing a piece of liver with 1 mm stainless steel beads at 30 Hz for 5 minutes in methanol + 0.1% acetic acid. The organic liver extract (20 μL) was dried down under vacuum and resuspended in 20 μL 0.1 M acetate buffer (pH 5.5) and deconjugated according to the manufacturer’s recommendation as previously outlined. Stable isotope dilution HPLC with LC-MS/MS was used for quantification of levels of 4-HPAA in plasma, liver, and osmotic pump contents following necropsy. The d6(methyl)-isotopologue of 4-HPAA was used as an internal standard (CDN Isotopes; D-7842). LC-MS/MS analyses were performed using an AB SCIEX Q-Trap 4000 triple quadrupole mass spectrometer equipped with an electrospray ionization source operating in negative ion mode. 4-HPAA was monitored using multiple reaction monitoring of precursor and characteristic product ions as follows: *m/z* 151.2 **→** 107.0 for 4-HPAA; *m/z* 157.2 **→** 113.0 for d6-4-HPAA. Mass spectrometry parameters were as follows: ions spray voltage –4200 V, ion source heater temperature 400°C, source gas 1 20 psi, source gas 2 30 psi, and curtain gas 20 psi. Nitrogen gas was used for the nebulizer, curtain, and collision gas. The HPLC system consisted of 4 binary pumps (LC-20 AD), autosampler operating at 10°C (Nexera X2 SIL-30AC), and controller (CBM-20A) (Shimadzu Scientific Instruments Inc.). Chromatographic separations were performed on a reverse-phase column (Kinetix XB-C18, 2.6 μm, 150 mm × 4.6 mm ID; Phenomenex, 00F-4496-E0). Mobile phase A was 5 mM ammonium acetate, and 0.1% acetic acid in water and mobile phase B was 0.1% acetic acid in methanol/acetonitrile (9:1; v/v). Samples were injected (10 μL) onto the column equilibrated in 100% A, and separated using a gradient as follows: 0–4 minutes 0% B, 4–20 minutes 0%–60% B, and 20–23 minutes 60%–100% B. Flow rate was programmed as follows: 0.3 mL/min. Samples were introduced to the mass spectrometer for analysis from 0–6 minutes.

### RNA sequencing in mouse tissues.

RNA was isolated via the RNeasy Plus Mini Kit (Qiagen) from mouse liver following the manufacturer’s protocol. RNA samples were checked for quality and quantity using the Bio-analyzer (Agilent). RNA-SEQ libraries were generated using the Illumina mRNA TruSEQ Directional library kit and sequenced using an Illumina NovaSeq 6000 (both according to the manufacturer’s instructions). RNA-Seq was performed by the University of Chicago Genomics Facility. Raw sequence files were deposited in the Sequence Read Archive (accession number SRP288008 under BioProject PRJNA670463). Paired-end 100 bp reads were controlled for quality with FastQC (v0.11.9, https://www.bioinformatics.babraham.ac.uk/projects/fastqc/). Concatenation of flow cells and alignment to the *Mus musculus* reference transcriptome (NCBI GRCm38.p6 transcript annotations downloaded August 2020) were performed in Galaxy (https://galaxyproject.org/) (45). Reads were aligned using the Kallisto pseudo-alignment (v0.46.0.4, https://pachterlab.github.io/kallisto/) (46) with standard parameters using Galaxy (45). Pseudo counts were loaded into R (http://www.R-project.org/; R Development Core Team, 2015), and DESeq2 (47) (v.1.28.1, https://bioconductor.org/packages/release/bioc/html/DESeq2.html) was used to perform differential expression (DE) analysis on genes with 10 counts per sample with α set to 0.05. *P* values were adjusted using the Benjamini-Hochberg method, and genes with a *P* value of less than 0.05 were considered statistically significant. Heatmaps were generated of top 50 differentially expressed transcripts using pheatmap (48) and RColorBrewer (49).NMDS analysis was performed using the plotMDS function of edgeR (50) using the top 500 DE genes as sorted by log_2_ fold change. Pathway analysis on the top 100 DE genes was performed using Metascape (51). The data discussed in this publication have been deposited in NCBI’s Gene Expression Omnibus (52) and are accessible through GEO (accession GSE159775; https://www.ncbi.nlm.nih.gov/geo/query/acc.cgi?acc= GSE159775).

### Plasma AST and ALT.

Plasma AST and ALT were measured after 2 weeks of intraportal delivery of either saline or 4-HPAA and compared with single-housed mice using the Sekisui Diagnostics Kit for AST and ALT measurement (part nos. 319-30 and 318-30, respectively) following the manufacturer’s protocol.

### Hematologic analysis.

To quantify WBCs, RBCs, and platelets, 50 μL of blood was collected from the tail vein into an EDTA-coated capillary tube before transfer into a new 0.6 mL tube containing 5 μL 0.1 M EDTA. Next, the sample was diluted with 150 μL sterile-filtered 3% bovine serum albumin in PBS. The sample was then analyzed on an ADVIA 120 Hematology System (Siemens Healthcare). Cell counts were determined after correcting for the dilution factor.

### Statistics.

Statistical analysis for more than 2 groups was performed using a 1-way ANOVA with Tukey’s multiple comparisons test using Prism 8.4.3 (GraphPad). For AST and ALT measurements, *P* values were calculated using Brown-Forsythe and Welch ANOVA with Dunnett’s multiple comparisons test. *P* values of less than 0.05 were considered significant. For comparisons between 2 groups, an unpaired 2-tailed *t* test was performed using Prism 8.4.3 (GraphPad). For the metabolic cage experiment, statistical analysis for energy expenditure was calculated using the generalized linear model with total body weight as a covariate, whereas ambulatory and locomotor activity were analyzed by ANOVA in CalR v1.2 (53), where data are shown as the mean ± SEM.

### Study approval.

All procedures in mice were reviewed and approved by the Cleveland Clinic Lerner Research IACUC and performed in accordance with relevant guidelines for rodent microsurgery.

## Author contributions

DO, LJO, and JMB planned the project, designed experiments, analyzed data, and wrote the first draft of the manuscript. FA, JC, and ZW helped design experiments and provided useful discussion directing collaborative aspects of the project. DO, LJO, KF, WM, AH, IC, BD, and ZW either conducted mouse experiments, performed biochemical workup of mouse and human tissues, analyzed data, or aided in manuscript preparation. All authors were involved in the editing of the final manuscript. The order of co–first authorship was determined by listing the last names alphabetically.

## Supplementary Material

Supplemental data

Supplemental Data Set 1

Supplemental Video 1

Supplemental Video 2

Supplemental Video 3

Supplemental Video 4

Supplemental Video 5

Supplemental Video 6

## Version 1 05/10/2021

Electronic publication

## Version 2 05/10/2021

Supplemental videos were added.
