# Supplementary figures and images for: A surgical method for continuous intraportal infusion of gut microbial metabolites in mice
Source: JCI Insight. 2021 May 10;6(9):e145607. doi: 10.1172/jci.insight.145607 (PMC8262340; doi:10.1172/jci.insight.145607)

● SH ● Sham ● NS

**A**

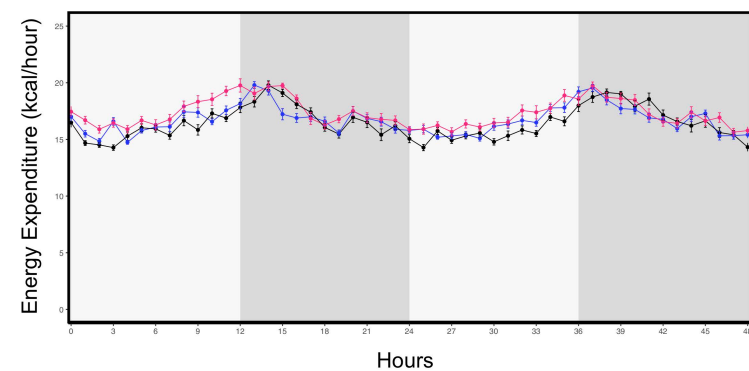

**B**

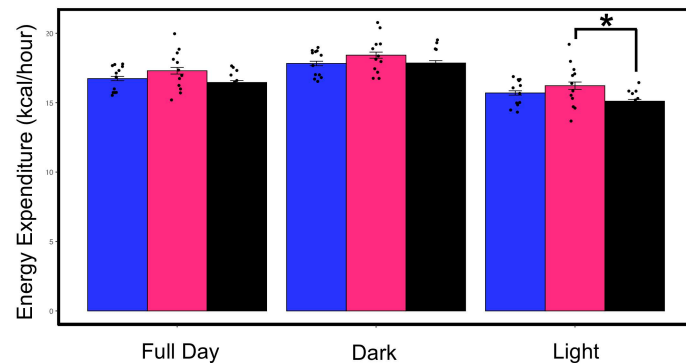

**C**

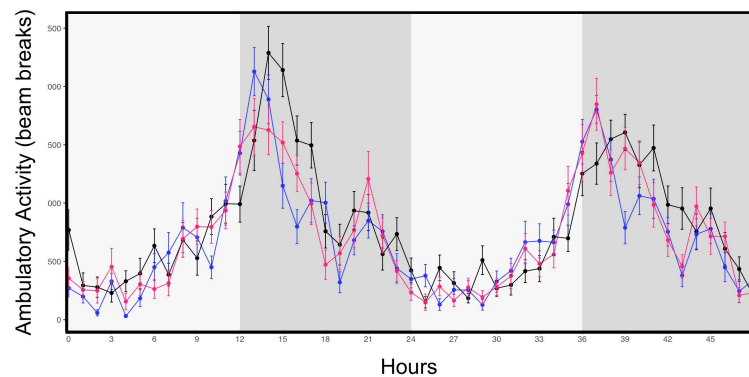

**D**

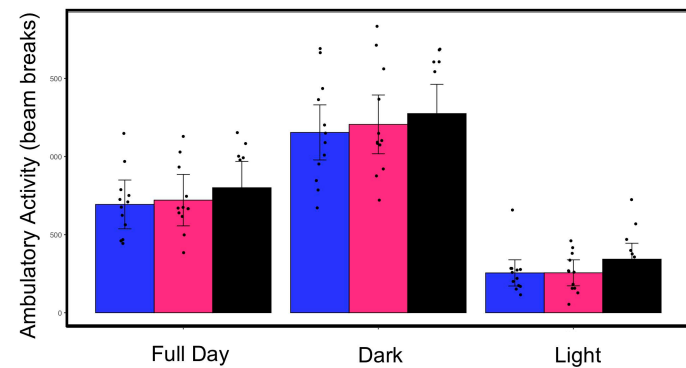

**E**

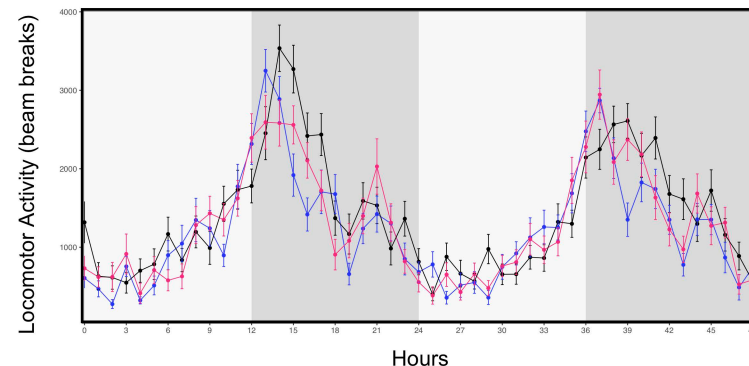

**F**

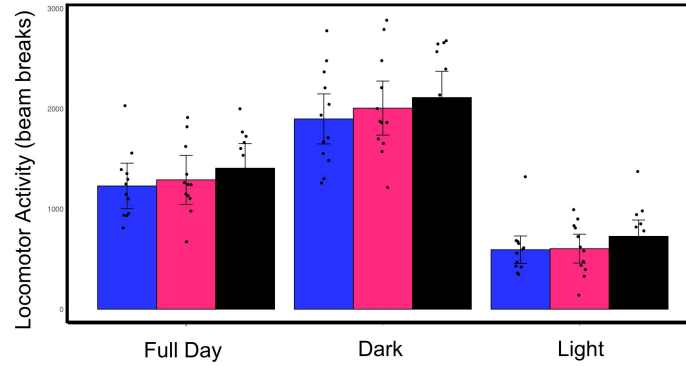

**A**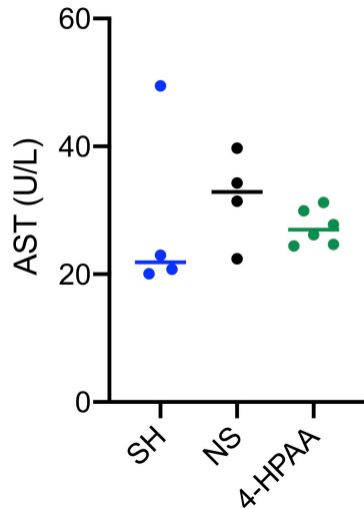**B**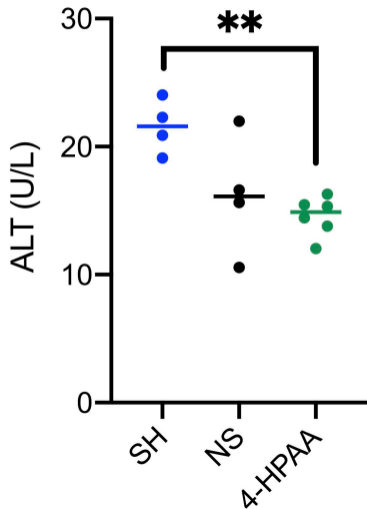

Supplement: Supplemental data [file jciinsight-6-145607-s307.pdf]
